# Changes in medical students´ and anesthesia technician trainees´ attitudes towards interprofessionality – experience from an interprofessional simulation-based course

**DOI:** 10.1186/s12909-022-03350-6

**Published:** 2022-04-13

**Authors:** Veronika Becker, Nana Jedlicska, Laura Scheide, Alexandra Nest, Stephan Kratzer, Dominik Hinzmann, Marjo Wijnen-Meijer, Pascal O. Berberat, Rainer Haseneder

**Affiliations:** 1 Technical University of Munich, TUM School of Medicine, Klinikum rechts der Isar, TUM Medical Education Center, Munich, Germany; 2 Technical University of Munich, TUM School of Medicine, Klinikum rechts der Isar, Department of Anaesthesiology and Intensive Care, Munich, Germany

**Keywords:** Simulation-based education, Attitudes, Medical students, Anesthesia technician trainees

## Abstract

**Background:**

Interprofessional simulation based education (IPSBE) programs positively impact participants' attitudes towards interprofessional collaboration and learning. However, the extent to which students in different health professions benefit and the underlying reasons for this are subject of ongoing debate.

**Methods:**

We developed a 14-h IPSBE course with scenarios of critical incidents or emergency cases. Participants were final year medical students (FYMS) and final year anesthesia technician trainees (FYATT). To assess attitudes towards interprofessionalism, the University of the West of England Interprofessional Questionnaire was administrated before and after the course. Using focus group illustration maps, qualitative data were obtained from a subcohort of the participants (*n* = 15).

**Results:**

After the course, self-assessment of communication and teamwork skills, attitudes towards interprofessional interactions and relationships showed comparative improvement in both professions. Attitudes towards interprofessional learning improved only in FYMS. Qualitative data revealed teamwork, communication, hierarchy and the perception of one’s own and other health profession as main topics that might underlie the changes in participants’ attitudes. An important factor was that participants got to know each other during the course and understood each other's tasks.

**Conclusions:**

Since adequate communication and teamwork skills and positive attitudes towards interprofessionality account to effective interprofessional collaboration, our data support intensifying IPSBE in undergraduate health care education.

**Supplementary Information:**

The online version contains supplementary material available at 10.1186/s12909-022-03350-6.

## Background

Interprofessional learning activities are increasingly implemented in various fields of healthcare education. In the context of anesthesia and perioperative patient care, effective interprofessional teamwork is of fundamental importance. It has been shown that poor teamwork in perioperative or other acute care settings is associated with increased errors [1], increased morbidity [2] and mortality [3]. Conversely, improved patient safety outcomes are reported after implementation of (interprofessional) team training programmes [4–6].

The goal of interprofessional educational (IPE) programs is not only to improve knowledge and skills in a specific area, but also to improve attitudes towards interprofessional learning and collaboration. The importance of healthcare providers’ positive attitudes towards collaboration as prerequisite for developing good interprofessional team work behavior and thus provide safe, high quality health care has been highlighted [7–10]. Simulation-based education can provide a realistic and safe learning environment which allows learners to experience and understand the consequences of their actions without danger to the patient. Kolb´s (1984) 'learning from experience' theory is often considered as theoretical background for simulation-based education [11]: In this model, the concrete, personally meaningful experience and its reflective observation play important roles in the experience-based learning process. Simulation-based education is known to improve self-efficacy for effective teamwork performance [12] and safety culture [13]. Interprofessional simulation-based education (IPSBE) programs improve teamwork behavior [14, 15] and team-based attitudes [16–19].

Although it is known that IPSBE programs have beneficial effects on participants' attitudes towards interprofessional collaboration and interprofessional learning in general [16, 20], the degree, to which students in different health professions benefit and the underlying reasons of the effects are debated. For example, there is both evidence that benefits after interprofessional educational interventions vary among professional groups [21, 22] or are comparable in magnitude [23–25]. The aim of this research project is to gain insight into the effects of an interprofessional simulation course with regard to attitudes towards interprofessional collaboration and learning and the reasons why these effects occur. For this purpose, we used both a quantitative and qualitative approach. The quantitative part was designed to answer the question of how strong the benefit of the interprofessional simulation course was among the different groups of participants. The qualitative part aimed to gather deeper knowledge and understanding of how these effects occurred and what insights the students gained during the course. The results of this study should help to identify the aspects of interprofessional education that contribute to the learning effect and thus to the development of effective interprofessional courses, which in turn would lead to improved patient safety.

## Methods

The study was reviewed and approved from an ethical and legal perspective by the ethics committee of the Technical University of Munich School of Medicine (registration numbers: 199/16S and 396/18S). Participation in the study was voluntary, and written informed consent was obtained from the participants before starting the data collection.

This was a single-center, quasi-experimental quantitative study designed as a pretest–posttest without a control group, combined with a qualitative study in order to gain a deeper understanding of the results of the quantitative analysis.

### Educational Intervention

We developed an interprofessional simulation-based education course, that mainly addressed crisis resource management principles [26, 27], but included also hands-on sessions (see below). The course consisted of four 3.5-h sessions distributed over four weeks. From July 2015 to December 2018, the course was delivered at six different dates in a standardized manner, with a total of 38 final year medical students (FYMS) and 38 final year anesthesia technician trainees (FYATT). Most of the FYMS had ‘anesthesiology’ as their final year elective discipline (*n* = 33, 87%). FYMS were invited to join the module on a voluntary basis, whereas for FYATT, participation was obligatory.

Unlike nurse anesthetists or anesthesiologist assistants, who administer anesthesia with a high level of autonomy in many countries, in Germany, the duties of anesthesia technicians are limited to assisting physician anesthesiologists in the perioperative period. Becoming an anesthesia technician requires a three year vocational training, which includes theoretical classes and rotations to different operating room areas. In Germany, during their final year in medical school, students pass through three sixteen-week rotations in the disciplines of surgery, internal medicine and an elective discipline. During this ‘practical year’, final year medical students observe and perform clinical activities with varying degrees of supervision within routine clinical settings.

The courses took place in the Medical Training Center of the Technical University of Munich. This training center is equipped with a virtual operation theatre, virtual patients’ rooms of a general hospital ward and a critical care unit, debriefing rooms, diverse computer-operated simulation manikins (Human Patient Simulator, CAE Healthcare; HAL S1000, Gaumard Scientific; Resusci Anne Simulator, Laerdal Medical) and audio video equipment.

Each session was conducted by four instructors, who operated the simulation manikins and facilitated the debriefing sessions, which followed each simulation scenario. The professional backgrounds of the instructors were anesthesiologists (*n* = 2), nurse anesthesist (*n* = 1) and anesthesia technician (*n* = 1). All instructors had several years’ experience in simulation team training. The simulation scenarios included critical incidents or emergency cases in the operating room or emergency department (an example case is presented in Additional File 1). In each simulation scenario, two FYMS and two FYATT participated actively, while all other participants observed the action via the audio video system. In addition to the scenarios and debriefings, a teaching session on the principles of crisis resource management, hands-on sessions on equipment preparation for emergency cases and airway management and a final reflection focusing on how to incorporate the learning experience into clinical practice were included in the course. Additional File 2 outlines the course structure.

In the debriefing sessions, the principles of crisis resource management (i.e., effective communication, team leadership, resource utilization, problem-solving, situational awareness) as well as other team-based competencies (e.g. shared mental model, role clarity, flattened hierarchy, speaking up) were addressed. The debriefing sessions were guided by interprofessional instructor teams consisting of one physician and one nurse anesthesist or anesthesia technician. They acted as facilitators to identify participants' performance gaps during the scenarios, to explore the root causes and frames of the participants underlying the performance gaps and to close the performance gaps by focusing on relevant principles in the particular situation [28, 29].

### Quantitative outcome measures

To assess changes in attitudes towards interprofessionality, we used the German version of the University of the West of England Interprofessional Questionnaire (UWE-IPQ) [30–33]. This questionnaire was chosen since it assesses health professionals' attitudes towards different aspects of interprofessionality including self-assessment of communication and teamwork skills, which are important learning outcomes of simulation-based programs focussing on crisis resource management. The UWE-IPQ consists of the four subscales ‘Communication and Teamwork’, ‘Interprofessional Learning’, ‘Interprofessional Interaction’, and ‘Interprofessional Relationships’ with each subscale containing eight or nine statements scoring on either a four- or five-point Likert scale. The ‘Communication and Teamwork’ subscale (nine items) appraises the respondents' self-assessment of communication and teamwork skills, the ‘Interprofessional Learning’ subscale (nine items) explores the respondents' attitudes towards IPE, the ‘Interprofessional Interaction’ subscale (nine items) appraises how the respondents perceive the quality of interprofessional interaction between other health care professionals, and the ‘Interprofessional Relationships’ subscale (eight items) appraises how the respondents perceive the quality of their *own* relationships with colleagues from their own and other profession [33, 30]. There is evidence of satisfactory to high reliability for the UWE-IPQ, with Cronbach’s alphas reported to be 0.76, 0.84, 0.82, and 0.71 for the four subscales [32, 33]. Concurrent validity was determined for the subscales ‘Communication and Teamwork’, ‘Interprofessional Learning’, and ‘Interprofessional Relationships’, with correlation coefficients reported to be 0.85, 0.84, and 0.72 [32, 33]. Validity of the subscale ‘Interprofessional Interaction’ is supported by qualitative data from students’ interviews on perceptions towards interprofessional interaction [32]. The entire questionnaire is published in 33. The paper-based UWE-IPQ was completed by the participants both before and after the course.

Additionally, demographic data and previously completed professional trainings were inquired about.

### Data collection and statistical analysis of the quantitative data

For each subscale, the selected options on the items were coded numerically (1–4 or 1–5) and summed up after recoding the reverse coded items. This resulted in sum scores with minimum 9 and maximum 36 points on the ‘Communication and Teamwork’ subscale, mininum 9 and maximum 45 points on the ‘Interprofessional Learning’ and ‘Interprofessional Interaction’ subscales, and mininum 8 and maximum 40 points on the ‘Interprofessional Relationships’ subscale, with lower values reflecting better attitudes or perceptions on each subscale. A unique code provided by the participants allowed us to link the respective pre-test and post-test questionnaires.

Since a hierarchical design was used (repeated measurements of participants´ scores nested within different modules), the data were analyzed using linear mixed-effects models with a compound symmetric covariance structure. Dependent variables were the sum scores on each subscale of each individual. In the model, ‘timepoint’ (pre versus post) and ‘professional background’ (FYMS versus FYATT) were entered as fixed factors. As a random factor, ‘course number’ was defined. If the effect of a fixed factor was significant, a post hoc pairwise comparison was performed. To test, whether changes in the dependent variables over different timepoints differed between groups (FYMS versus FYATT), ‘timepoint’ by ‘professional background’ interaction terms were included in the regression model. For group comparisons on demographics, Fisher's exact test and the Mann–Whitney-Test were used. P-values < 0.05 were considered to indicate statistically significant differences.

Effect sizes were calculated using Cohen’s d analysis. Cronbach’s alpha was used to test the internal consistency of each subscale. Unless otherwise stated, the results are presented as estimated means or estimated mean differences (between-timepoint differences or between-group differences of between-timepoint differences) with 95% confidence intervals (CI). IBM® SPSS® Statistics version 25 and GraphPad Prism 6 were used for the statistical analysis.

### Data collection and analysis of the qualitative data

A qualitative approach was used to gain further insight into the situations and experiences during the course, which may have changed participants’ attitudes towards interprofessionality. We decided to use focus group illustration maps (FIM) as a special method for group interviewing and analyzing data [34]. FIM combines the realization of focus groups and the data analysis method of knowledge mapping. FIM allow a flexible and economical procedure to give relatively prompt feedback to course developers. As data collection and evaluation run at the same time, without the need for a literal transcription, it offers a timesaving method. Using FIM, complex focus group discussions can be summarized, structured and graphically presented, as described elsewhere [34]. We developed the guideline for the focus group sessions on the basis of the UWE-IPQ. It consisted of the following four questions:

1. *Remember the course and situations, when you became active in a team… what happend there?*
2. *How did you experience collaboration with FYMS or FYATT?*
3. *What do you think. Now, after the course, which challenges and opportunities are there if people from different health professions work together?*
4. *Now, after the course, how would you shape the relationship to future colleagues from your own and from other professions?*

These questions were framed in order to deepen the four UWE-IPQ subscales. Our questions during focus groups where phrased to give participants the opportunity to describe specific interprofessional situations during the course. We asked about their thoughts, feelings and judgements, and about challenges and successful moments.

All participants who attended the course in November/December 2018 were invited to join the focus group interviews. One participant missed the last session and could therefore not take part in the focus group interviews. For the interviews, the participants were divided into three subgroups (*n* = 6 FYMS, *n* = 4 FYATT, and *n* = 5 FYATT), and three focus group interviews were held simultaneously right after the last session. We decided to conduct focus group interviews within each profession in order to create a safe atmosphere for the participants to talk freely. Each interview team consisted of one moderator and one co-moderator; none of them were involved in the teaching sessions in order to allow students to speak freely. All moderators had prior experience in moderating a focus group. Additionally, every moderating team attended an internal training on how to moderate a focus group one week prior to the interviews. In keeping with FIM, during focus group interviews the moderator was responsible for directing the discussion and asking questions. The co-moderator’s job was to write down the participants' arguments on a flipchart. The statements were summarized in such a way that each participant could recognize their own point of view. The moderator read out all recorded arguments before introducing the next topic. In this way, participants were encouraged to recapitulate the discussion and withdraw or add new arguments. Thus, a consensual validation of the discussion points with regard to the completeness of the presentation took place. The visualization of the arguments formed the basis for the creation of the maps and thus the first step of the data analysis. Each topic of the interview guideline was discussed for about 15 min. The whole interview lasted between 60 and 80 min. The focus groups were audiotaped in order to prepare the maps and reconstruct the arguments.

We started our analysis by representing the qualitative data graphically. LS und NJ analyzed the flipcharts and audiotapes for each question and focus group. First, the arguments made during the focus groups with the FYMS were transferred into the mind mapping software Mindjet MindManager 2019 (Corel Corporation). Next, the recordings of the focus group discussion with the FYMS were listened to and further details were added to the arguments. Listening to the recordings also served the purpose of better assessing the weighting of the individual contributions. Thus, a tree map was made, which showed all the arguments of the FYMS concerning the UWE-IPQ’s first subscale. This step was reproduced for every subscale and for the FYATT. The two FYATT focus groups where combined in one tree map. During the first step we also organized the arguments into subgroups and moved them to the subscale that we found most fitting. We then had eight tree-maps in total; Four reflecting the FYATT opinion and four reflecting the FYMS opinion. To further condense the data, we combined the FYATT and FYMS opinions and arguments for each subscale. Differences of opinion by the two professions were color-coded. During this procedure we again organized and summarized the arguments. This led to four final maps.

To further evaluate the data, we developed a three-step framework: 1. Does the argument describe or asses a situation during the course? 2. Does the description or situation concern interprofessionality? 3. Does this possibly entail a shift of the UWE-IPQ results? Each argument that did not fit this framework was deleted (e.g. arguments which did not refer to course situations). This framework and the UWE-IPQ questionnaire resulted in four condensed maps. Additional File 3 illustrates the process of qualitative data analysis, Additional File 4 shows one of the condensed maps as an example.

For analytical rigor, all steps were undertaken by at least two researchers and discussed within the entire research team. Finally, NJ worked with the last four maps and identified topics which showed up on the maps recurrently. Those themes are described in the Results section.

Following the principles of convergent-parallel mixed-method approach [35], the quantitative and qualitative data were collected and analyzed concurrently but independently. In the following step, the results of the two data sets were merged by comparing, contrasting, and synthesizing the individual results. In the course of interpreting the merged results, the researchers discussed to what extent and in what ways the results converged, diverged, related to each other, and provided a more comprehensive understanding.

## Results

### Results of the quantitative data

A total of 76 participants attended one of the six dates of the course between July 2015 and November 2018. Table 1 shows the demographic data.

**Table 1:** Demographic characteristics of the participants

	**All**	**FYMS**	**FYATT**	**P**
Male (n, %)	27 (36)	14 (37)	13 (34)	> 0.999^a^
Age (mean ± SD)	25.3 ± 4.4	27.4 ± 4.3	23.1 ± 3.3	< 0.001^b^
Median (IQR)	25 (5)	26 (3)	22 (3)	
Mininum, maximum	20, 47	24, 47	20, 34	
Completion of other professional training (n, %)	21 (28)	7 (18)	14 (37)	0.123^a^

*n* number, *SD* standard deviation, *IQR* interquartile range, *FYMS* final year medical students, *FYATT* final year anesthesia technician trainees

^a^Fisher´s exact test

^b^Mann-Whitney-Test

Of the 76 participants, 27 were male. The participants ranged from 20 to 47 years of age, with a median age of 25. 21 of the participants had completed another professional training before entering medical school or anesthesia technician school. The most commonly completed professional trainings were emergency paramedic [8] and physician's assistant [6]. Cronbach's alphas for the ‘Communication and Teamwork’ subscale, the ‘Interprofessional Learning’ subscale, the ‘Interprofessional Interaction’ subscale, or the ‘Interprofessional Relationships’ subscale were 0.67, 0.79, 0.68, or 0.81, respectively, indicating moderate to good internal consistency for all subscales.

**Fig. 1:**
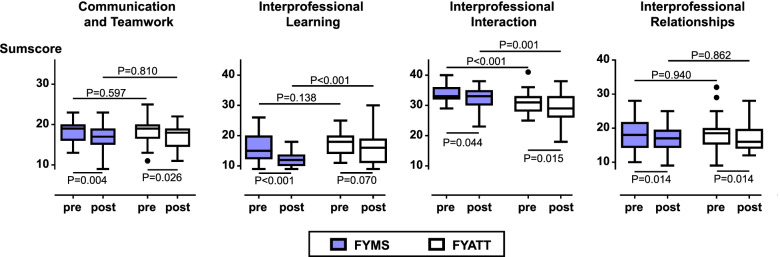
Boxplots illustrating the results of final year medical students (FYMS) and final year anesthesia technician trainees (FYATT) on the four subscales of the University of the West of England Interprofessional Questionnaire. After the training (post), significant improvements in both FYMS and FYATT were seen on the subscales ‘Teamwork and Communication’, ‘Interprofessional Interaction’ and ‘Interprofessional Relationships’. On the subscale ‘Interprofessional Learning’, a significant improvement was only seen in FYMS. On the subscale ‘Interprofessional Interaction’, scores of FYATT were consistently better compared to scores of FYMS. Minimum and maximum sum scores are 9 and 36 points in the ‘Communication and Teamwork’ subscale, 9 and 45 points in the ‘Interprofessional Learning’ and ‘Interprofessional Interaction’ subscales, and 8 and 40 points in the ‘Interprofessional Relationships’ subscale. Lower values reflect better attitudes or perceptions in each subscale

Figure 1 and Table 2 show the main quantitative outcome measures.

**Table 2:** Quantitative outcome measures

Subscale	Group	Estimated mean score t_0_ [95% CI]	P (FYMS versus FYATT) t_0_	Estimated mean score t_1_ [95% CI]	P (FYMS versus FYATT) t_1_	Estimated mean difference (t_1_-t_0_) [95% CI]	P (t_0_ versus t_1_)	Effect size Cohen´s d (t_0_ versus t_1_) [95% CI]	Between group difference of between timepoint difference [95% CI] P
**Communication and Teamwork**	FYMS	18.41 [16.82, 20.01]	0.597	16.96 [15.36, 18.56]	0.810	-1.45 [-2.40, -0.50]	0.004	0.35 [-0.10, 0.80]	-0.29 [-1.56, 0.98] 0.651
	FYATT	18.84 [17.74, 19.95]		17.13 [16.01, 18.24]		-1.72 [-2.60, -0.84]	0.026	0.51 [0.05, 0.97]	
**Interprofessional Learning**	FYMS	15.90 [14.68, 17.11]	0.138	12.40 [11.10, 13.70]	< 0.001	-3.49 [-4.93, -2.05]	< 0.001	0.90 [0.43, 1.37]	2.12 [0.07, 4.18] 0.043
	FYATT	17.35 [15.11, 19.59]		15.96 [13.71, 18.20]		-1.40 [-2.91, 0.12]	0.070	0.24 [-0.21, 0.69]	
**Interprofessional Interaction**	FYMS	33.86 [32.78, 34.93]	< 0.001	32.50 [31.34, 33.66]	0.001	-1.35 [-2.67, -0.04]	0.044	0.39 [-0.06, 0.85]	-0.46 [-2.39, 1.46] 0.631
	FYATT	30.87 [29.21, 32.53]		29.06 [27.40, 30.72]		-1.81 [-3.25, -0.37]	0.015	0.40 [-0.06, 0.85]	
**Interprofessional Relationships**	FYMS	18.28 [16.44, 20.12]	0.940	16.83 [14.98, 18.68]	0.862	-1.45 [-2.59, -0.32]	0.014	0.30 [-0.15, 0.76]	0.01 [-1.56, 1.58] 0.989
	FYATT	18.44 [16.41, 20.48]		17.02 [14.98, 19.06]		-1.43 [-2.54, -0.31]	0.014	0.27 [-0.19, 0.72]	

*CI* confidence interval, *FYMS* final year medical students, *FYATT* final year anesthesia technician trainees, *t*_*0*_ before training, *t*_*1*_ after training

Minimum and maximum sum scores are 9 and 36 points in the ‘Communication and Teamwork’ subscale, 9 and 45 points in the ‘Interprofessional Learning’ and ‘Interprofessional Interaction’ subscales, and 8 and 40 points in the ‘Interprofessional Relationships’ subscale

With respect to the participants' self-assessment of communication and teamwork skills, a significant improvement in both the FYMS and FYATT was observed after the training. Analysis of the interaction term (time x group) revealed no significant between group (FYMS versus FYATT) difference when comparing the improvement of self-assessed communication and teamwork skills (*P* = 0.651).

The estimated mean scores on the ‘Interprofessional Learning’ subscale were not significantly different between the FYMS and FYATT before the training. Analysis of the interaction term (time x group) revealed a significant between group difference when comparing the FYMS with FYATT (*P* = 0.043): The estimated mean scores of the FYMS significantly improved after the training, whereas the estimated mean scores of the FYATT did not.

The estimated mean scores on the ‘Interprofessional Interaction’ subscale of the FYMS were significantly better compared to that of the FYATT, both before and after the training. In both groups, the estimated mean scores significantly improved after the training with no significant between-group difference (P for interaction (time x group) = 0.631).

The estimated mean scores on the ‘Interprofessional Relationships’ subscale did not significantly differ between the FYMS or FYATT neither before nor after the training. Compared to baseline values, estimated mean scores on the ‘Interprofessional Relationships’ subscale of both the FYMS and FYATT significantly improved after the training with no significant between-group difference (P for interaction = 0.989).

### Results of the qualitative data

We identified four main themes in the qualitative data: (1) teamwork, (2) communication, (3) hierarchy and (4) the perception of one’s own and other health profession.

#### (1) Teamwork

With respect to the perception of teamwork in the scenarios, we discovered two contradictory perspectives. Several interviewees evaluated teamwork as successful. In particular, they valued the willingness of participants to cooperate, the exchange of knowledge and showing respect in dealing with colleagues. On the other hand, some of the participants perceived the teamwork during the scenarios as insufficient. These interviewees criticized the participants’ rigid focus on their own tasks, instead of working together towards a common goal. The FYMS complained about the poor collaboration by the FYATT. They felt the FYATT had been more concerned with folliwing medical students’ orders and did contributory work without reflecting on the situation critically and questioning decisions. The FYMS also complained about the passivity of the FYATT in the decision-making process concerning further procedures and expressed their wish for more ideas and proposals as well as more objections and opposition from the FYATT. In their opinion, the FYATT did not take the opportunity to work on equal terms with medical students and they showed little initiative despite their extensive experience in this area. Thus, medical students found themselves forced to take the lead, a task which they managed “surprisingly well” in their own opinion.

*I actually would have expected a bit of a different dynamic there, I also didn't know what the education level of a third year FYATT is and I just assumed that they probably know more, now specifically about anesthesia and the situation in the operating room, and then I was actually quite surprised that they fell instantly and often into sort of a co-worker role, and I don't think I've received a single word of opposition or anything like that in any of the scenarios, although I'm sure I haven't always made the right decisions. (…) Yes, and therefore I would actually have expected more objection or more personal initiative and there was, in my opinion, surprisingly little. (FYMS)*

The FYATT, for their part, complained that the medical students did not keep an eye on them. They were missing a person whom they could refer to. Overall, the interviewees agreed that teamwork improved during the course.

Further, the interviewees talked about how they envision future collaboration and relationships with colleagues from other health professions. They stressed the importance of good interpersonal relationships and strong team spirit. The open and respectful way of dealing with each other, the willingness to help and showing gratitude were outlined as key aspects of successful collaboration. The course illustrated to them the importance and necessity of teamwork for patient safety and, at the same time, made them aware of the existing lack of collaboration between different health professionals. The cooperation at an early stage of the study and professional training was identified by the participants as an opportunity to facilitate teamwork.

*I think it has now become clear that we simply have to work together, and that this hierarchy has to be discarded, because it's not about who has the better professional group, this simply is all about the patient, who probably could become endangered. (FYATT)*

#### (2) Communication

Communication was the second main theme. Both groups of interviewees perceived communication in the scenarios as insufficient. Especially at the beginning of the course, communication was perceived as “difficult”. Unlike the FYATT, the FYMS identified the lack of knowledge about other health professionals, about their background, expertise and professional skills as a factor which made communication difficult. According to the FYMS, the poor knowledge about other medical professions poses a problem insofar as there is no guarantee that what is said would be understood properly and the other person comes to similar conclusions. As a result, at the beginning of the course, medical students mainly communicated with other medical students. Their poor knowledge about the expertise of other health professionals also required more intense communication to structure the situation at the beginning of a scenario.

*Well, first I had kind of a problem dealing with it. What do the FYATT know, and what's their professional background. Then I also asked myself what can they already do? And that was right at the beginning. As a consequence I then communicated more with the other FYMS, because I just knew their background, I knew that if I tell them this and that, they would come to similar conclusions and then would evaluate it similarly to me, or at least would be able to evaluate it. (FYMS)*

However, both groups of interviewees referred to the pleasant and friendly atmosphere, which enabled the cooperative communication in scenarios. The FYATT especially valued the opportunity to reflect on the scenario and express their opinion self-consciously in contrast to real practice, in which they often do not dare to speak out. One FYATT referred to the experiences in the clinic and explained:

*Well, I often don't dare to say anything because I think to myself, yes, if it is wrong, somehow I will be blamed for it. Or they'll remember it: Oh, she actually got that wrong! And this somehow makes me afraid to say anything because I think to myself, talking is silver, silence is golden. Before I say anything wrong, and that this is then attached to me for a long time, I prefer not to say anything. (FAYTT)*

After getting to know each other and with the growing experience of working with each other, the communication improved. This intense communication facilitated mutual learning, sharing of knowledge and allowed both sides to benefit from each other’s expertise.

Overall, the interviewees identified the lack of communication between the different health professionals as a major challenge. Insufficient communication increases stress, affects collaboration – and thus patient care – negatively.

#### (3) Hierarchy

The interviewees commented on their perception of hierarchy in the scenarios. They pointed out the absence of hierarchy between the FYMS and FYATT. From the perspective of the FYATT, it was even possible to change roles with the FYMS and take on the leadership of the team. The collaboration on an equal footing facilitated the active involvement of the participants in the process, allowed them to make decisions autonomously and gave them the feeling of being useful.

*I judged it beneficial that the FYMS were also so open with us and did not look down on us but really worked very well with us and always involved us, and there was somehow no hierarchy. (FYATT)*

Moreover, the interviewees commented on their general attitude towards hierarchy in medicine. Interestingly, we identified two contradictory perspectives on hierarchy. On the one hand, both groups of interviewees outlined thinking and acting hierarchically in medicine as a big challenge for collaboration. The FYMS attempted to explain the “mindless subservience” of the FYATT in the scenarios. The FYMS referred to the hierarchy and mentioned the lower position of FYATT as the reason why FYATT did not dare to object and simply followed the medical students’ instructions. For their part, the FYATT also referred to the distribution of roles in the scenarios and explained their own restrained attitude through their role as nursing staff. The FYATT sought to inhabit their idea of a nurse who, in their opinion, has to follow physicians’ instructions and orders. Thus, they expected more direction from the medical students, who were the physicians in the scenarios, and complained about the lack of instructions.

*Well, to be honest I sometimes enjoyed relying on the FYMS. I thought to myself: Okay, they are now playing their role as a doctor, that’s what they want to be then, and so actually listened to their instructions. And yes, to be honest, in fact I relied on them a little bit to much and left the task of thinking to them, and preferred to leave them to do their own thing. (FYATT)*

Moreover, it can be assumed that the FYATT transferred this hierarchical thinking and acting from their real-world practice to the scenarios. During the interview, the FYATT discussed the challenges in collaboration with the physicians at the work setting extensively. They outlined the hierarchical structure and described how the fear of not being taken seriously because of their lower position in the medical hierarchy, the anxiety about saying something wrong and being reprimanded for it led them to not take the initiative and remain passive. More respect for one other and the acceptance that working areas are different but equal were outlined as key to changing the hierarchy in medicine. The interprofessional course and thus the opportunity to get to know each other, was seen as a chance to facilitate this change.

On the other hand, the interviewees emphasized the importance of the hierarchy for successful teamwork. Both the FYMS and FYATT liked the fact that the hierarchy bestows structure and creates order. They warned against over-fraternization and the need for distance to maintain the hierarchical order. Correspondingly, several of the FYATT criticized the lack of clarity concerning the hierarchical order or rather the difficulty of maintaining the hierarchy in the scenarios.

#### (4) The perception of one’s own and other health profession

The interviewees expressed their perceptions of their own and other health profession. It was noticable that their self-perception and their perception of the other medical profession, along with the associated expectations of themselves and towards the other health profession, impacted their own action and influenced overall collaboration. The lack of knowledge about the other medical profession revealed prejudices that often led to false expectations and uncertainties. Medical students assumed that the FYATT had practical experience and know-how in anesthesia and expected them to show more initiative during scenarios. The FYMS were surprised that the FYATT did not take the initiative and only performed contributory work. For their part, the FYATT overestimated the medical students’ professional knowledge and skills, which was stated as a reason for the FYATT’ lack of initiative.

Moreover, the FYMS saw their expectations of each other and of themselves, as well as their need to prove themselves, as a challenge for the collaboration. The FYATT complained that the FYMS focused excessively their own tasks and offered the FYATT too little supervision. Several of the FYATT felt under pressure to demonstrate their practical know-how, which led them to perceive the scenario situations as stressful.

The interviewees agreed that collaboration enabled them to get to know each other better, contributed allaying prejudices and changed their perception of their counterparts. The knowledge gained contributed to the dissolution of the misconceptions about the expertise of other health professionals, led to adjusting expectations and thus to avoiding conflicts.

*I believe that because now we know a little more about what they can do, and they now also know what we can do, it increased mutual understanding. (FYMS)*

The FYATT learned that physicians could be friendly, that they are not omniscient and also “only human”. For their part, the FYMS learned to value the practical knowledge of the FYATT. Furthermore, the experiences with the medical students strengthened the self-confidence of the FYATT, encouraged them to take more initiative and communicate more openly.

*There are a few things that we do not know, and there are also some things the students do not know, things WE can do. And that's how it is, this collaboration is always quite right then, because everyone comes up with something that the other can't do. If YOU can't do it, then I may be able to do it. (FYATT)*

## Discussion

By combining quantitative and qualitative research approaches, the present study aimed to investigate the effects of an interprofessional simulation training on FYMS´ and FYATT´ attitudes towards interprofessional collaboration and learning and to understand why these effects accrue.

We were able to demonstrate that after a 14 h IPSBE training, self-assessment of communication and teamwork skills improved in both FYMS and FYATT, as did attitudes towards interprofessional interaction and interprofessional relationships. An improvement of attitudes towards interprofessional learning, however, was only seen in FYMS.

A positive impact of interprofessional simulation training on attitudes towards interprofessional learning and collaboration has been shown within different settings and for different professions [16, 21, 36, 20, 23, 17, 25], and our study confirms these data. Both FYMS and FYATT showed better attitudes after the training in almost all domains under investigation.

A profession dependent difference was only observed within the domain of interprofessional learning. The FYMS' attitudes towards interprofessional learning significantly improved after the training, whereas those of FYATT did not. On this point, the quantitative results contradict the qualitative results of our study. In the focus groups, both FYMS and FYATT expressed an open-minded attitude towards interprofessional learning after the course. The course was described by the interviewees as a chance to facilitate their teamwork and communication skills. The participants appreciated the opportunity to gain knowledge about the other health profession, get to know future colleagues and overcome prejudices and thereby facilitating mutual understanding, enhanced communication and teamwork which contributed to the change of hierarchical thinking and acting. The medical students emphasized the lasting impact of the course and reported paying more attention to FYATT in real-world surgery situations several days after the course. Both the FYAM and the FAYTT expressed their clear wish for more interprofessional courses at an early stage of their studies and professional training. One possible explanation for the differences between quantitative and qualitative results could be the fact that in the focus group we did not explicitly ask about attitudes towards interprofessional learning. We made this decision to avoid desirable responses and identify the interviewees’ attitudes without influencing their opinions.

Interprofession differences in the impact of simulation training on attitudes towards IPE have also been shown recently in a study by Leithead et al.: They showed that IPSBE improved attitudes towards collaboration and IPE in medical students and nurse anesthesia students, but not undergraduate nursing students [21]. In another study, before and after a resuscitation training, nursing students scored higher in the subscale ‘roles and responsibilites’ of the Readiness for Interprofessional Learning Scale compared to medical students, which was attributed to nursing students’ previous IPE experiences and/or more clinical exposure [22]. In contrast, other studies show that interprofessional simulation trainings improve participants’ perceptions of IPE in both medical and nursing students [23, 24] or medical, nursing, and respiratory therapy students [25] to a comparable degree. A recent study found no differences when comparing final-year medical and nursing students’ perceptions in domains like ‘team cohesion’ or ‘power distance’ after an interprofessional simulation training [37]. We speculate that the causes of the discrepancies of these results to our results are most likely multifactorial. Such factors might include different educational interventions, different professional groups and different local (e.g., country specific) mentalities of the participants.

In our study, we saw that both the FYMS and FYATT expressed comparatively negative opinions about interprofessional interaction, with FYMS’ scores being more negative compared to that of the FYATT. The qualitative results allow explanations for these negative assessments. The lack of knowledge about the other health professions with the simultaneous existence of stereotypes as well as the negative experiences in hierarchical work practice may have contributed to FYMS’ and FYATT’ negative attitudes towards interprofessional interaction. The more negative scores of the FYMS compared to the FYATT could be explained by the often tense relationship with the nursing staff, which was underlined by the medical students in the focus groups.

A non-positive view about collaborative working relationships between different professions has also been reported by other studies for students from various healthcare professions on entry to pre-qualifying programs [32], as well as for first-year medical students [38]. It has been suggested that this negative view might be due to the exposure of individuals as students to practice settings [39], as well as by stereotypes recognized by students at the beginning of their professional career [38]. The negative view changed when individuals worked as qualified practitioners [30]. In our study, after the training, attitudes towards interprofessional interaction and relationships improved comparably in both professions, which may indicate that the training reduced stereotypical thinking. Our qualitative results enable a deeper understanding of this development. The challenges in collaboration with different health professionals were discussed extensively in the focus groups. As described above, hierarchy and the lack of knowledge about other health professions were outlined as a compounding factors. Thus, it may be assumed that the absence of hierarchy during the course that facilitated participants’ active involvement in scenarios was a positive experience of collaboration that led to a change of participants’ attitudes towards interprofessional interaction and relationships. Further, it was evident that the training provided the opportunity to get to know each other and gain knowledge about the other health profession. This contributed to breaking down prejudices, changed the perception of the counterparts and thus the attitude towards interprofessional interaction and relationships. An improvement in the perception of stereotypes has not only been shown after IPE programs that focus on communication [40] or roles and function of healthcare providers [41], but also after simulation trainings in high-acuity care settings [23].

To our knowledge, qualitative data on attitudes and perceptions of undergraduates in the context of interprofessional simulation training in high-acuity settings is limited to relatively few studies: After interprofessional resuscitation training, interview analysis of undergraduate medical and nursing students revealed perceived benefits for teamwork, communication and role perceptions [42, 22], as well as hierarchy issues as barriers to teamwork [22]. Qualitative data from another work, where final year medical, nursing and nursing anesthesia students participated in an emergency medicine interprofessional simulation training revealed learning outcomes in the domains of self-insight, stress management, understanding of the leadership role, insight into teamwork, and skills in team communication [43]. In a study with an interprofessional high-acuity multipatient simulation experience, nursing students reported a positive learning experience and the importance of collaboration and teamwork [20]. Nursing students, nurse anesthetist students, and medical students report communication, impact of debriefing and realism as the most beneficial effects of an IPSBE course in the operating room [16]. Undergraduate medical and nursing students reported on better realization of teamwork fundamentals, reconsideration of professional roles, and achievement of increased confidence after interprofessional emergency simulation training [44]. After a pediatric high-acuity simulation training, undergraduate medical and nursing students highlighted the improved clinical skills and the safe learning environment [18].

The qualitative results of our study are consistent with these findings and extend them by providing a more comprehensive understanding of some of these topics. Our analysis revealed four main themes that seem to play a role with regard to interprofessionality from the perspective of the interviewees: [1] teamwork, [2] communication, [3] hierarchy and [4] the perception of one’s own and other health profession.

In general, IPE programs improve the understanding of teamworking [45], and better insights into teamwork as a learning outcome after an interprofessional simulation training are reported for undergraduate final year medical students [43, 44], nursing students [20, 43, 44] and nursing anesthesia students [43]. Our results correspond with these findings identifying teamwork as central component of interprofessional collaboration. Concerning the perception of teamwork, we identified two contradictory perspectives. On the one hand, the interviewees referred to the open-minded attitude of their counterparts and evaluated teamwork during scenarios as successful. On the other hand, several interviewees criticized participants’ rigid focus on their own tasks and evaluated teamwork during scenarios as insufficient. The ambivalent perception of teamwork by our participants may be explained by the existing lack of knowledge about the other health professions and thus by misconceptions about the expertise of other health professions. Furthermore, echoing Bradley et al.’s findings [22], we were able to determine the existing hierarchical structures with defined roles and responsibilities as a reason for the conflicting perceptions of collaboration. Overall, our interviewees agreed that teamwork improved during the course. Thus, in line with previous research [22, 42] the positive impact of training on teamwork appeared to be confirmed.

Communication skills are of fundamental importance for interprofessional teamwork [46, 47], and interprofessional simulation trainings are able to convey this importance to medical and nursing students [43]. Improved communication after IPSBE courses has been reported in different settings and for different professions [16, 19, 43, 48]. Our qualitative findings reflect these findings and provide further insight into the reasons for it. The interviewees in our study criticized the poor communication in scenarios. The above mentioned lack of knowledge about the other health professionals’ expertise was identified as a factor which undermined smooth communication. Thus, getting to know each other – to gain knowledge about other health professionals and their background, expertise and professional skills – along with the experience of working with each other were identified as factors that contributed to the improvement of communication. Overall, in accord with the literature, our participants considered our course as a chance to improve their communication skills.

In general, hierarchies may provide certain positive aspects for teamwork in terms of defining roles and responsibilities, facilitating rapid decision making and establishing order [49, 50]. With regard to hierarchy, we identified two contrasting perspectives. On the one hand, participants agreed that thinking and acting hierarchically in medicine affects collaboration between different medical professionals negatively. These findings are in line with a body of literature, that identifies hierarchy as a barrier to effective interprofessional communication, e.g., in the setting of operating room teams [51, 52] or teams in emergency departments [53, 54]. On the other hand, the interviewees also saw value in hierarchy in providing structure, and emphasized its great importance for successful teamwork. These findings are consistent with studies that describe positive aspects of hierarchy e.g. by anesthesiologists in the context of critical events [52]. Hierarchy and strong leadership are thought to be of fundamental importance in ensuring the effectiveness of resuscitation teams [55, 56]. It can be presumed our participants' conflicting attitudes towards hierarchy in medicine could be traced back to the legal accountability that goes hand in hand with physicians' decision-making power and responsibility [57]. Whether this hypothesis holds and whether there is a causal relationship between physician decision-making power and accompanying responsibility and attitude toward hierarchy should be explored in subsequent studies.

The perception of one’s own and other health profession constituted the fourth main theme in our data. We saw that the interviewees entered the course with certain perceptions of themselves and of the other medical professionals. These perceptions with their associated expectations greatly impacted the participants' own actions and thus overall collaboration. The importance of understanding one´s own role and the roles of other team members for effective team work has been highlighted by other studies [58, 59]. A positive impact of interprofessional simulation training on the understanding of nurses' roles in patient care and decision making has been reported previously [20], and the simulation training in our study led the participants to gain more respect for other professions [60]. As with the statements given by the participants in our study, improved understanding of professional roles has been reported in medical, nursing and pharmacy students after IPSBE programs [22, 44, 60, 61].

### Study limitations

Our study has important limitations that have to be considered. Qualitative data was obtained only from a subcohort of the participants. However, separate analysis of the quantitative data of this subcohort revealed largely comparable results compared to the quantitative data of the entire cohort (data not shown). Additionally, the method-related waiver of the transcripts for the focus groups can be interpreted as further limitation.

With respect to the FYMS, there might have been a selection bias, since their participation was on a voluntary basis. This might have led to participating FYMS being those who were more motivated and interested in IPE and simulation-based training.

The fact that the study was conducted at one medical school, in combination with the relatively small sample size without a priori sample size estimation and the non-random sample selection has implications for the ability to generalize the findings.

Additionally, since there was no control group, conclusions on the causality should be drawn with caution: It might be possible that participants' responses after the training partly resulted from other experiences of their ongoing professional training and/or activity. However, this threat is diminished by the rather short period of time between the pre- and posttest. Additionally, the results of the qualitative part of the work describing the effects of the course and attributing these benefits directly to the course experience allow us to suggest causality. Nonetheless, to draw definitive conclusions on the causality, a study with a more rigorous research methodology such as a randomized controlled trial would be necessary.

## Conclusion

In our study, we found that after a 14 h interprofessional simulation-based training, self-assessed communication and teamwork skills as well as attitudes towards interprofessional interaction and interprofessional relationships improved in both FYMS and FYATT. The study identified the lack of communication between the different health professionals as a major challenge for effective interprofessional teamwork. Lack of knowledge about the other health professions might be a central problem that undermines smooth communication between different health professionals. The course induced the participants to reflect on the image of one’s own and other health professions and triggered the dissolution of prejudices and misconceptions about the expertise of other health professionals resulting from the lack of knowledge about their knowledge and skills. The study underlines the double character of hierarchical thinking and acting and suggests that while hierarchy can be detrimental to working relationships if too inflexibly adhered to, it provides necessary and reassuring order and structure. These aspects are critical for effective interprofessional collaboration, which in turn results in improved patient safety and quality of care. Thus, the data favors an integration of interprofessional simulation training in undergraduate health care education.

## Supplementary Information

**Additional file 1. **Example Case – Anaphylactic Shock.

**Additional file 2. **Course structure.

**Additional file 3. **Analysis of qualitative data.

**Additional file 4. **Teamwork with the other health profession.

## Data Availability

The data that support the findings of this study are available from the corresponding author (RH) upon reasonable request.

## Abbreviations

CI: Confidence intervals
FIM: Focus group illustration maps
FYATT: Final year anesthesia technician trainees
FYMS: Final year medical students
IPE: Interprofessional education
IPSBE: Interprofessional simulation-based education
UWE-IPQ: University of the West of England Interprofessional Questionnaire
