# Supplementary material for: Changes in medical students´ and anesthesia technician trainees´ attitudes towards interprofessionality – experience from an interprofessional simulation-based course
Source: BMC Med Educ. 2022 Apr 13;22:273. doi: 10.1186/s12909-022-03350-6 (PMC9006475; doi:10.1186/s12909-022-03350-6)
Supplement: Supplementary file 1 — Additional file 1. Example Case – Anaphylactic Shock. [file 12909_2022_3350_MOESM1_ESM.pdf]

Additional file 1

Example Case – Anaphylactic Shock

Short synopsis: Patient Mr. Nothinger gets surgery on inguinal hernia with spinal anesthesia and develops fulminant anaphylactic reaction upon administration of cefuroxime.

Time for case: 15 to 20 minutes

Time for debriefing: 45 to 60 minutes

Setting: Operating theatre

Distribution of roles: one resident anesthetist, two anesthesia technicians, one attending anesthetist

Equipment: Standard equipment operating theatre, simulation manikin, defibrillator, emergency cart, infusion pumps, surgical drapes, sterile towels, cefuroxime, patient file, premedication sheet, anesthesia protocol "Mr. Nothinger"

Patient: Mr. Nothinger (80 kg, 175 cm), needs repair of inguinal hernia (left side), no relevant pre-existing illnesses

Preparation:

- Simulation manikin in reverse Trendelenburg position with spinal anesthesia, oxygen via nasal cannula, lying IV access with antibiotic connected, spontaneously breathing, awake, monitoring connected, vital signs: heart rate 80/min, blood pressure 100/60 mmHg, peripheral oxygen saturation 97%
- Patient file, premedication sheet, anesthesia protocol "Mr. Nothinger" lying on the anesthesia ventilator
- Operating field covered with sterile towels for inguinal hernia surgery, wound care material positioned

Situation: Patient with spinal anesthesia, cefuroxime is to be applied, resident anesthetist at the head end, one anesthesia technician within shouting distance in front of the door, attending anesthetist and second anesthesia technicians outside, reachable by phone

First phase of the scenario: After start of cefuroxime infusion the patient develops dyspnoea, brochospasticity and diffuse urticarial rash. Manikin changes vital parameters in terms of a severe anaphylactic shock (including bronchospasticity). Participants have to recognize anaphylactic reaction (possible differential diagnosis e.g. high/total spinal block) and stop the allergen supply.

How the scenario unfolds:

- Mr. Nothinger continues to decline with worsening respiratory distress, hypotension and tachycardia within two to four minutes.

- Improvement can only be achieved when epinephrine and second line therapeutics (antihistamines, steroids, IV fluids, second large caliber IV access) are given.

- The resident on site should call the attending anesthesist and second anesthesia technician early in the scenario.

- Significant delay of epinephrine administration results in deterioration to pulseless electrical activity and will require cardiopulmonary resuscitation.

- Depending on the performance of the participants, pulmonary situation may only slowly improve, so that intubation might be necessary.
