# Supplementary figures and images for: Changes in medical students´ and anesthesia technician trainees´ attitudes towards interprofessionality – experience from an interprofessional simulation-based course
Source: BMC Med Educ. 2022 Apr 13;22:273. doi: 10.1186/s12909-022-03350-6 (PMC9006475; doi:10.1186/s12909-022-03350-6)

## Additional file 2 – Course structure

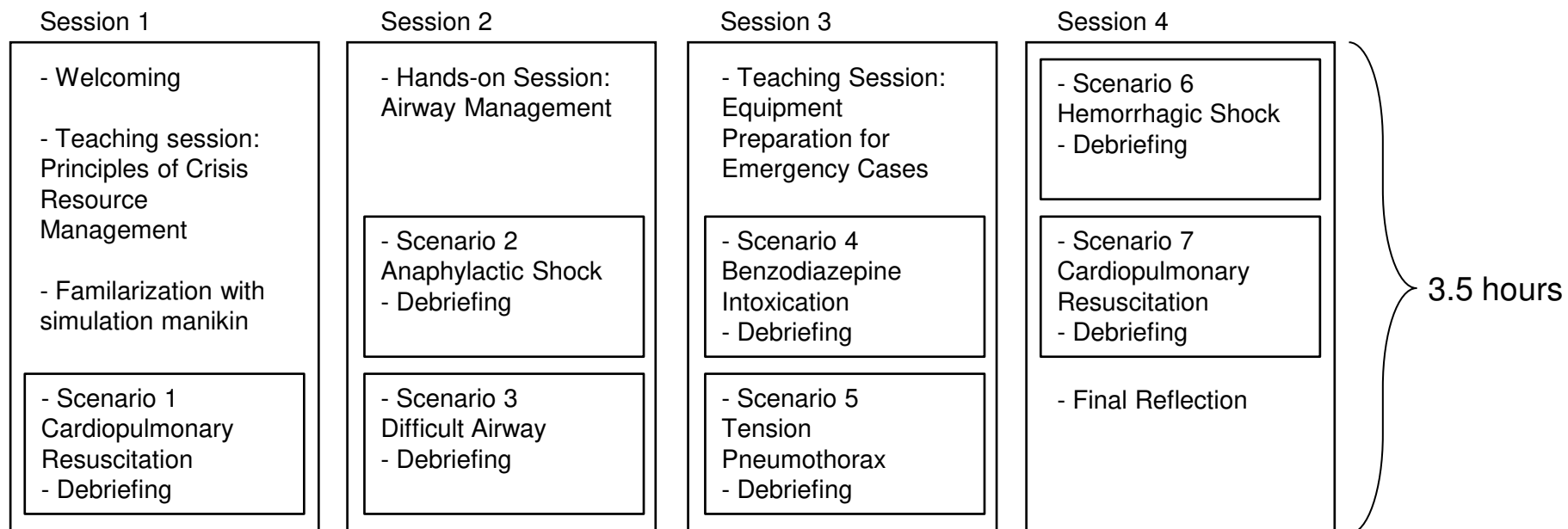

Supplement: Supplementary file 2 — Additional file 2. Course structure. [file 12909_2022_3350_MOESM2_ESM.pdf]

Additional file 3

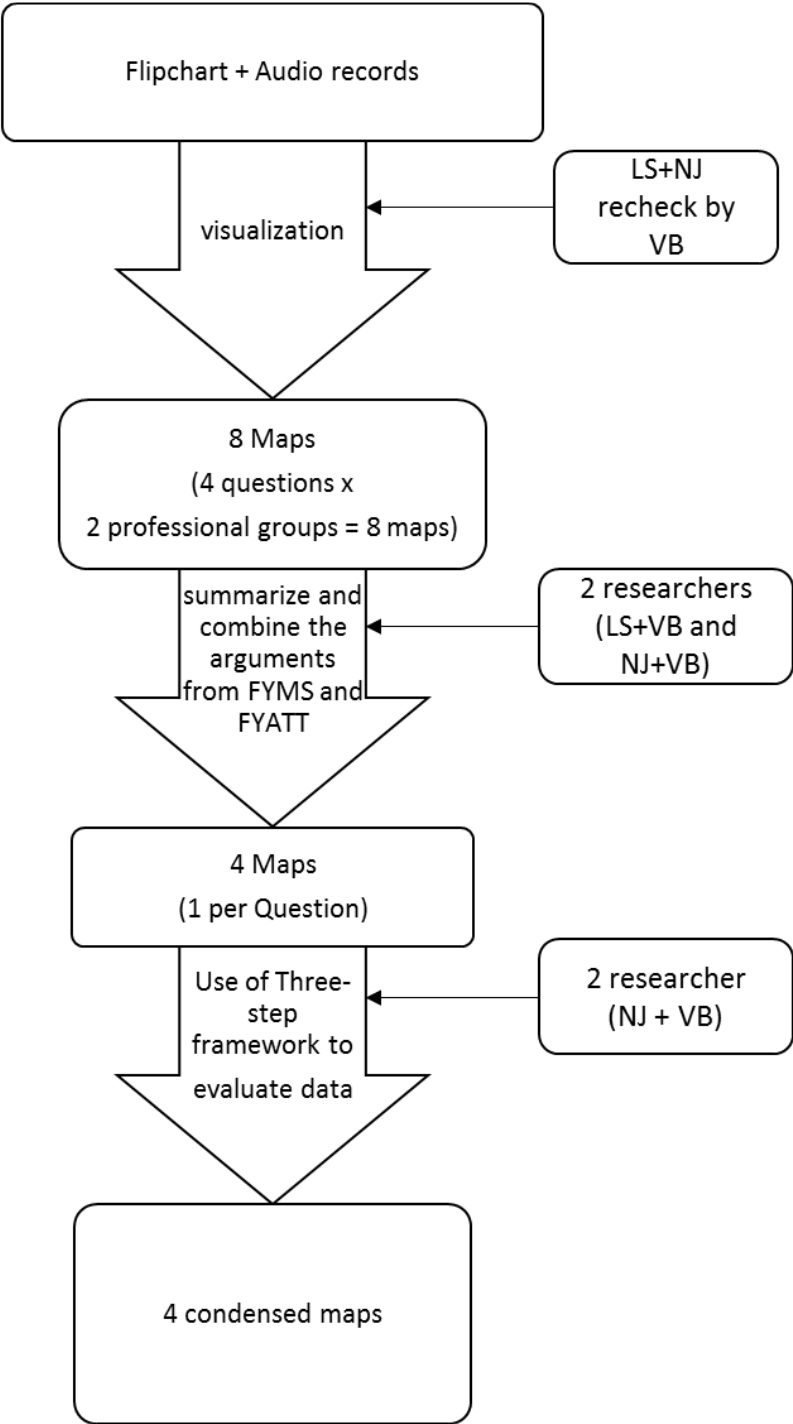

Supplement: Supplementary file 3 — Additional file 3. Analysis of qualitative data. [file 12909_2022_3350_MOESM3_ESM.pdf]
