# Supplementary material for: Changes in medical students´ and anesthesia technician trainees´ attitudes towards interprofessionality – experience from an interprofessional simulation-based course
Source: BMC Med Educ. 2022 Apr 13;22:273. doi: 10.1186/s12909-022-03350-6 (PMC9006475; doi:10.1186/s12909-022-03350-6)
Supplement: Supplementary file 4 — Additional file 4. Teamwork with the other health profession. [file 12909_2022_3350_MOESM4_ESM.pdf]

# Additional file 4

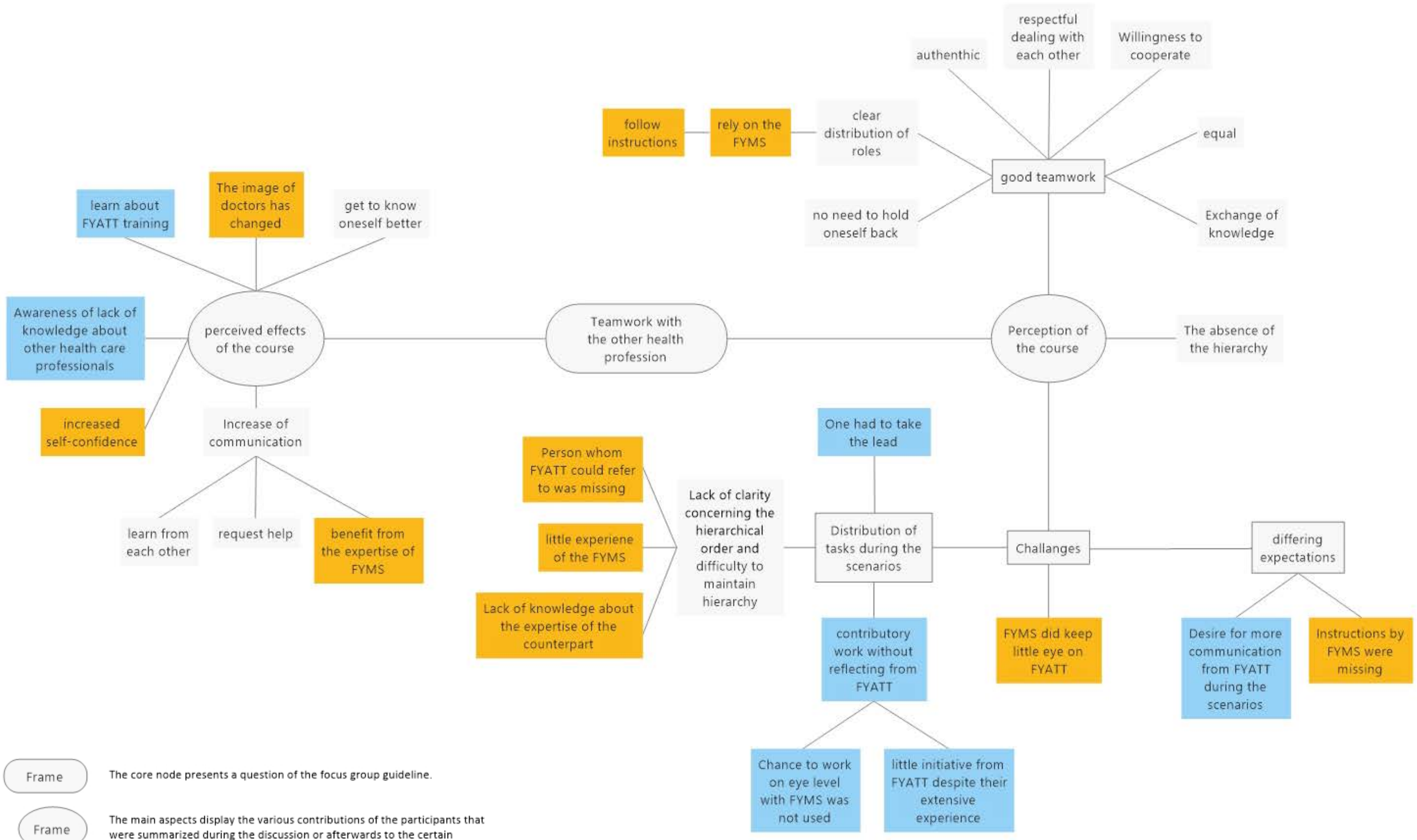


| | |
|---|---|
| Frame | The core node presents a question of the focus group guideline. |
| Frame | The main aspects display the various contributions of the participants that were summarized during the discussion or afterwards to the certain clusters and given a fitting name. |
| Frame | The subaspects represent the different topics within the main aspects. |
| Frame | The individual statements that relate to the topics. |
| Color | The statements of the FYYMS are shown in blue. |
| Color | The statements of the FYATT are shown in orange. |
